# RETRACTION: Hydroxysafflor Yellow A Inhibits TNF‐*α*‐Induced Inflammation of Human Fetal Lung Fibroblasts via NF‐*κ*B Signaling Pathway

**DOI:** 10.1155/ecam/9841764

**Published:** 2026-04-07

**Authors:** Evidence-Based Complementary and Alternative Medicine

RETRACTION: S. Liu, Y. Wang, H. Wen, X. Sun, and Y. Wang, “Hydroxysafflor Yellow A Inhibits TNF‐*α*‐Induced Inflammation of Human Fetal Lung Fibroblasts via NF‐*κ*B Signaling Pathway,” *Evidence-Based Complementary and Alternative Medicine* 2019, no. 1 (2019): 4050327, https://doi.org/10.1155/2019/4050327.

The above article, published online on 26 December 2019 in Wiley Online Library (https://wileyonlinelibrary.com), has been retracted by John Wiley & Sons Ltd. The authors requested a retraction due to data concerns and concerns that the data could not be reproduced. After further investigation, concerns were identified with the western blots presented in Figures 1 and 2.

More specifically:

• In Figure 1G, the IL‐6 panel appears to be duplicated with the β‐actin panel in Figure 7D of [1].
• In Figure 2C, the IL‐6 panel appears to be duplicated with the β‐actin panel in Figure 5B of [2].
• In Figure 2C, the β‐actin panel appears to be duplicated with the GAPDH panel in Figure 2C of [3].

As a result of the investigation, the data and conclusions of this article are considered unreliable.

The authors did not respond when notified of the retraction.
